# Evolution of RFID Applications in Construction: A Literature Review

**DOI:** 10.3390/s150715988

**Published:** 2015-07-03

**Authors:** Enrique Valero, Antonio Adán, Carlos Cerrada

**Affiliations:** 1 School of Energy, Geoscience, Infrastructure and Society, Heriot-Watt University, Edinburgh EH14 4AS, UK; 2 3D Visual Computing and Robotics Lab, Universidad de Castilla-La Mancha, Paseo de la Universidad, 4, 13071 Ciudad Real, Spain; E-Mail: Antonio.Adan@uclm.es; 3 Escuela Técnica Superior de Ingeniería Informática, Universidad Nacional de Educación a Distancia, Juan del Rosal, 16, 28040 Madrid, Spain; E-Mail: ccerrada@issi.uned.es

**Keywords:** RFID, construction, smart technologies, building

## Abstract

Radio frequency identification (RFID) technology has been widely used in the field of construction during the last two decades. Basically, RFID facilitates the control on a wide variety of processes in different stages of the lifecycle of a building, from its conception to its inhabitance. The main objective of this paper is to present a review of RFID applications in the construction industry, pointing out the existing developments, limitations and gaps. The paper presents the establishment of the RFID technology in four main stages of the lifecycle of a facility: planning and design, construction and commission and operation and maintenance. Concerning this last stage, an RFID application aiming to facilitate the identification of pieces of furniture in scanned inhabited environments is presented. Conclusions and future advances are presented at the end of the paper.

## 1. Introduction

The concept of a smart environment was proposed by Philips, and it appeared for the first time in the literature in 1999 [1,2]. A smart environment is defined as a place where different technological devices (sensors, readers, computers, *etc.*) are invisible and unobtrusive to the users, and it is easy and effortless to interact with them [3]. For example, an elemental proposal of a smart environment is a room where lights are turned on if luminosity is under a limit value and there are people inside who need this resource. This definition leads to several research lines related to automating the interactions human to human or human to machine. This philosophy supposes a significant change when it comes to interacting with technological devices. Traditional keyboards or mice are being replaced by other kinds of devices that are less demanding of the user (remote controls, wireless sensors, touch-sensitive screens, *etc.*).

One of the most extended and promising wireless non-contact systems is radio frequency identification (RFID) [4], a technology based on the exchange of information by means of electromagnetic signals. Because of its ability to identify and track objects, RFID is being used for diverse applications: aviation, construction and facility management, health, retailing, logistics or security, among others. A brief resume of RFID applications is given in the next paragraphs.

In the food industry, it is important to trace the history and the localization of products aiming to guarantee quality and security in the food chain. In [5], the author discusses the use of RFID systems in the cold chain logistics of fresh products. Mennecke and Townsend [6] propose an RFID system to determine the product provenance in the meat production industry. Furthermore, in consumer packaged goods management, several important companies require their providers to install RFID tags in pallets or boxes in order to improve the processes of storage, inventory and security.

In the field of healthcare, there exist works in which RFID technologies are used to manage different aspects of a hospital. Some tasks, such as blood transfusions, are controlled by means of RFID technologies [7] in order to find the correct blood bag for a specific patient. Amini *et al.* [8] propose the use of an RFID system to collect data related to the movements of trauma patients.

Tracking the position of components in a manufacture chain is a process commonly carried out by means of RFID technologies. For instance, in robots construction [9], the integration of an RFID device in the fabrication process allows obtaining information about the following tasks. Infineon Technologies, one of the largest semiconductor manufacturers in the world, has created an identification and localization system using RFID and ultrasound sensors aiming to improve the logistics in the wafer fabrication process [10].

Since the 1990s, RFID has been applied in the field of construction. As will be stated in the forthcoming sections, an important number of works has been published, and different reviews have been presented. The main objective of this paper is to present a new literature review improving the previous ones on several points:

Organization of the paper: The entire lifecycle of a building has been taken into account, from its conception (planning and design) to operation and maintenance once it is inhabited, also considering the construction and commission stage.

Extending the study framework: The paper is not only focused on the management of materials/resources and the construction site monitoring (as in [11]), but the processes before and after the construction procedure have also been tackled.

Current state-of-the-art: The most recent works in this field of research, which are not considered in previous reviews ([11–13]), have been included in the paper.

The document is organized as follows: Section 2 presents a brief introduction to RFID. In Section 3, certain applications of RFID in the initial stages of the constructive process are presented. Section 4 is devoted to showing the monitoring of construction sites and the tracking of resources by means of RFID technologies. In Section 5, some approaches regarding evaluation and maintenance are presented. Several works in which radio frequency identification is used to locate users and to navigate interiors are shown in Section 6. Section 7 tackles new lines of research, in which several technologies are combined with RFID. Finally, in Section 8, we discuss the different proposals and present the conclusions.

## 2. A Brief Introduction to RFID Technology

**Figure 1:**
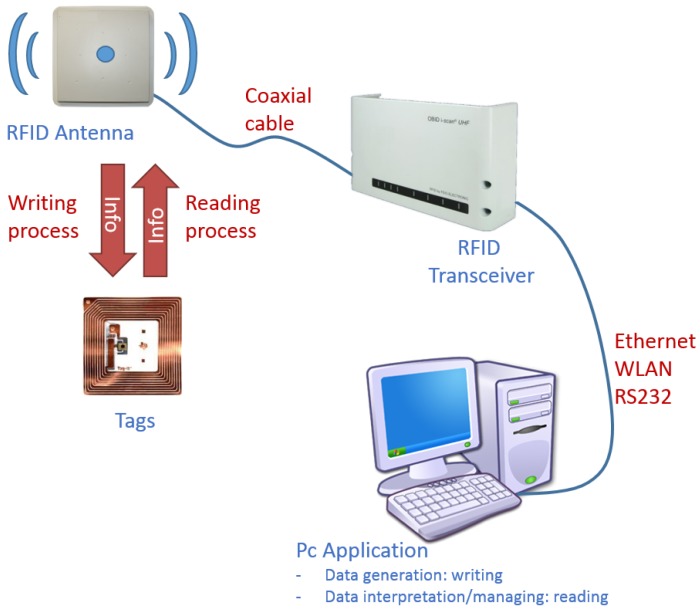
Example of an RFID system.

An RFID system (see Figure 1) is mainly composed of a transceiver (called the reader) connected to an antenna and a set of transponders or tags, where information is stored [14]. The transceiver communicates with a computer by means of an application, which manages the data stored in the tags.

Antennas establish the communication between the transceiver and transponders. Depending on the distance between the system and the objects to which the tags are adhered, there exist several kinds of antennas and tags. Table 1 shows the different operation bands and their field of action.

This paper aims to review different applications of RFID technologies in the field of construction. In a building under construction, we need to control some information related to components or even people, which are sparse in the environment. Therefore, distances are in the range of dozens of meters. As highlighted in Table 1, in most cases, the antennas used for tracking materials or workers are UHF antennas.

As mentioned before, the information exchanged by the RFID system is stored in devices called tags. The system's range of action is also influenced by the type of tags adhered to the objects. These devices contain two parts: an integrated circuit, which stores and processes the information, modulates the signal and collects power from the transceiver if necessary; and an antenna for transmitting and receiving the signal.

There exist three types of tags: active, passive and semi-passive [15,16]. Active tags incorporate the power supply and transmit the signal to the transceiver. On the other hand, passive tags acquire the required energy from the readers. Passive tags have a limited range of action (15 m), whereas active ones can be used for distances of up to 100 m. Finally, semi-passive tags can transmit, but backscattering is used. In addition to this, these tags need to be turned on by a signal. Table 2 shows the differences between the different kinds of tags.

## 3. RFID Technology in the Initial Stages of the Constructive Process: Planning and Design

Radio frequency identification technology demonstrates a great potential in the monitoring of constructive processes. Among these operations, a wide variety of them are carried out before the building work starts. On the one hand, several processes are related to the conception of different RFID systems aiming to facilitate the constructive process. On the other hand, one can consider the use of RFID in the production of different materials used in the building construction. This section provides different applications in these previous stages.

### 3.1. Planning the Implementation of RFID Systems in the Construction Industry

The environment and working conditions are very different for each constructive action. Therefore, a particular RFID system is implemented for each context. Materials are diverse according to the sort of construction; *i.e.*, weather is different depending on the location, and the chosen solution varies if works are carried out indoors, outdoors or buried.

As highlighted in the previous section, an RFID system is composed of a reader (or a writer-reader) device equipped with antennas and several tags. The storage capacity of the tags and their position in the scene, the type of device and its operation frequency depend on several parameters, such as: the need for modifying the tag contents and the privacy of the data, the distance of interrogation, the used materials or the portability of the reader [17]. The material of the components to which the tags are adhered could severely interfere with the operation of RFID; hence the importance of evaluating the influence of combining materials and RFID tags [18]. Metal surfaces can amplify the signal of an active RFID tag. However, steel is considered a major issue when it comes to using passive tags. As Jaselskis and El-Misalami stated in [17], if a passive tag is mounted on a metallic piece, it needs to be mounted around 1 cm from the surface. If some tags are placed closer, anti-collision procedures have to be considered. Furthermore, if there are electromagnetic sources working under a frequency similar to that of the system, special considerations should be made. A more in-depth study about the interferences of metal surfaces on passive tags has been carried out by Mo and Zhang [19]. In this work, the authors state that a tag placed at a distance of λ/4 (being λ the wavelength) allows the tag antenna to receive more energy to power the chip. One of the more recent works on using RFID in construction scenarios has been developed by [20], in which the authors investigate the feasibility of BAP (battery-assisted passive) and passive RFID tags in metal-dense scenarios.

Taking into consideration this issue related to metallic surfaces, new tags have been designed to be mounted on metallic objects [21], reaching a similar read range when mounted on metallic surfaces and working in free space.

Another particular environment is presented in [22]. In this work, the need for accurate records of an underground infrastructure aims to establish a buried locating system by means of RFID technology. Table 3 illustrates the main objectives of the above-mentioned works.

### 3.2. Construction Components Manufacturing and Supply Chain

Once the building construction is planned, different materials are moved from fabrication to the job site. In the last few years, RFID technology has been gaining importance in this supply of components, the decisions about the use of RFID systems in supply chains being an important issue [23].

In 1995, Jaselskis *et al.* [24] proposed to incorporate RFID technology into delivery, billing and quality control for concrete. The steps of concrete mixing, loading time and delivery location would be monitored and notified to the job site and test lab. More recently, Moon and Yang [25] presented a new monitoring system for concrete pouring, storing the data generated during this process and converting them into valuable information for production and quality control.

In order to save time in the construction operations, precast components are being used more and more to raise buildings. The fabrication of this kind of element can be inspected and managed by means of RFID systems, as shown in [26], where a precast production management system is developed to examine incoming materials and the production and logistic processes.

Other parts that are prefabricated before building erection are pipe spools. In every industrial project, constructors install an important amount of unique pipe spools. These components are associated with a critical and costly process going through design, fabrication, delivery, storage, installation and inspection. This is a long supply chain whose surveillance could be subject to many problems. An RFID application [27] can solve these possible issues by means of the automation of a tracking process for the control of parts in the factory.

The above-mentioned tasks involve the transportation of materials from the factory to the construction site. The control of these delivery jobs is therefore considered as a part of the constructive process. Grau *et al.* [28] study the shipping and receiving of steel constructive components, which are transported in a trailer. The authors have analysed different kinds of tags, the bed conditions and the truck speed. Related to this conveying analysis, the mileage of the vehicles is an important factor to consider, aiming to improve the efficiency of these trips and to reduce carbon dioxide emissions. This objective can be achieved by means of a radio frequency identification system helping to reduce the *CO*_2_ emissions from transportation and to increase the vehicle loading rate [29].

Different RFID applications for materials manufacturing and transport are compared in Table 4.

## 4. Construction and Commission Control Using RFID Technologies

In the course of building activities, thousands of materials and a crew of workers equipped with tools and vehicles are permanently changing their position in the workplace. The control of these movements helps improve the productivity in building erection, and the safety of workers can be increased. The resource management at construction sites is not a new challenge, involving the organization of the storage area and the control of deliveries. According to Thomas *et al.* [30], the benefit/cost ratio of an effective material management could be up to 5.7, showing the clear advantages of paying attention to material management.

Nonetheless, a manual supervision and recording of these activities is a time-consuming and expensive task. Bjork [31] presents the idea of computer-integrated construction (CIC) at the end of the 1980s, looking for the automatic control of the entire construction process by means of the replacement of manual drafting for computers.

### 4.1. Tracking Systems: Materials and Resources

#### 4.1.1. Materials

RFID technology offers the chance to locate materials in construction applications at a fast update rate and at an accuracy varying from one to a few meters. Since the 1990s, the possibility of taking into account RFID technologies for managing critical materials, equipment and vehicles has existed [24].

Calculating the location or even the pose [9] of wireless sensors in a construction site can be really helpful to identify the materials located in the scene and to estimate their position. Several techniques have been developed in the last few years aiming at this objective, dealing with the improvement of the location accuracy [32]. One of these approaches consists of integrating a Global Positioning System (GPS) with RFID devices aiming to look for tagged objects in a real 3D scenario, improving the accuracy of outdoor RFID localization [33]. Other techniques aiming to estimate the position of materials at a job site are based on determining the proximity of an object with respect to other known locations [34]. Aiming to avoid the limitations of RFID and GPS-based systems, the combination of ultrasound technologies with an RFID system [35] can improve the positioning performance.

As mentioned in Section 3.2, prefabricated components have been used in construction in the last few decades. These structural elements are numerous and commonly scattered around the job site, making real-time monitoring of them truly complex. Structural steel components, such as beams, and precast concrete panels [36] are equipped with RFID tags in order to track their position in the construction area. The use of metal tags improves the robustness of the data acquisition process.

Great amounts of components enter the construction sites, but also an important quantity of residual soils need to be removed from this area. In most countries, these soils must be deposed in special sites for reusing or recycling. A couple of RFID readers equipped with cameras are installed in the job and disposal sites, and RFID tags are adhered to the windscreen of the trucks to control the correct movement of the waste material [37]. More recently, Zhang and Atkins [38] have presented a waste management system for construction based on RFID. In this work, RFID helps generate records about location, volume, weight and tracking of containers and vehicles, providing real-time decision support.

#### 4.1.2. Resources

Not only the materials, but also other resources, such as workers or equipment, have to be controlled in the construction site. On many occasions, the evaluation of works has to be carried out by a supervisor. However, there are many employees moving at the same time around the job site. Aiming to control the operations executed by workers, they can be equipped with RFID tags [39] that register the movements of the labour force and working time.

The poor quantity of tools or their misplacement in the workplace are related to undesirable interruptions. Workers have to look for the proper tool in the scene, this being a time-consuming task. In other construction scenes, the number of tools exceeds the necessary quantity to avoid delays. However, the addition of RFID tags to the equipment [40] can be a useful strategy to optimize the budget.

Cranes and excavators are dangerous vehicles that can cause accidents during the building process. In spite of the safety conditions in the construction scenes, thousands of workers are wounded every year. More precisely, vehicles and heavy equipment are one of the primary sources of fatalities in construction, accounting for nearly 36% [41]. The development of a personal warning system [42] can alert workers to the dangers of hazards using working area information. Wu *et al.* [43] have studied and classified the category and frequency of struck-by-falling-object accidents, controlling the position of materials and labour force by means of a ZigBee RFID system.

Table 5 presents the proposals mentioned in Section 4, indicating if the authors combine different technologies.

### 4.2. Construction Site Monitoring

Monitoring is a determining task to achieve an accurate measurement of progress in building sites. In addition, this control strategy helps manage labour, safety and equipment. All of these aspects are separately evaluated with the help of RFID systems, as shown in Section 4.1. However, there also exists a variety of works in which different methods are integrated to manage an overall project. For example, Yoon *et al.* [44] propose the control of every component in ordering, production, transportation, storing, installation and inspection jobs.

The continuous movement of materials and workers in the job site, together with the advances in the works, make the monitoring of resources in the construction environment truly complicated. RFID technologies are not able to control every process carried out in the workplace. However, the combination of several technologies can automate the monitoring systems, helping measure the progress of every process. Thus, laser scanning and photogrammetry can be used for site representation, RFID and barcodes for collecting actual working hours and modelling for information purposes and updating planned data [45]. The control of the status of materials in the job sites by means of RFID technology is a useful tool to show a 4D CAD model of the building under construction and to compare the planned model with the built one [46]. To visualize the construction progress in real time, other authors [47] propose the integration of building information management (BIM) models and augmented reality. They suggest the use of an RFID system to track different components.

In one of the last publications in this field, Liu *et al.* [48] present a combination of technologies (RFID, GPS, PDA and GPRS ) that monitors the watering operations in the construction of earth-rock dams. Trucks are equipped with RFID tags that provide the position of the vehicles.

These works, leading to the combination of RFID with other technologies to monitor the construction sites, are compared in Table 6.

## 5. Operation and Maintenance of Buildings

The quality requirements in construction projects are factors to take into account, especially to detect possible defects, which entail an important economic impact. Between 5 and 10% of construction costs are due to the reconstruction of defective components detected during the building activities [49]. Most part of these defects is due to human factors, such as the inefficient supervision of the constructive process.

RFID technology has contributed to facilitating and automating the maintenance and evaluation tasks in buildings. One of the evaluation jobs carried out during the construction phase is the testing of the drainage systems. The control of drainage systems by means of balls equipped with RFID tags allows the evaluation, in a simultaneous manner, of several parts of the pipeline, reducing the operation time by 80% and the manpower by 50% [50]. Furthermore, the obtained results can be stored in RFID tags and PDA devices, reducing the human errors.

The lifecycle of a finished building is closely linked to its maintenance, either in new buildings or reconstructed or extended ones [51]. Several tasks are related to the periodic inspections of certain elements and their reparation or reposition if it is advisable. The management of the information linked to these works greatly improves with the addition of RFID technologies to the facility management field. Thus, the identification of the monitored components is carried out in an automatic way, reducing errors and operation time. The information of the status of each component, stored in RFID tags, can be useful to monitor the condition of the equipment in real time and to manage maintenance sequences [52].

Safety is still an extremely important factor to consider once buildings are inhabited, fires being one of the more frequent and feared accidents. Aiming to manage this kind of issue in a more effective manner, a set of RFID tags can then be used for storing information about the history and the condition of the extinguishers and valves [53]. This information will be also useful for further inspections and maintenance labour.

On some occasions, the fire cannot be controlled by the occupants, and the fire department then acts to avoid a serious disaster. In these cases, the automation of the fire rescue procedure can greatly reduce the delays and even save lives. Shiau *et al.* [54] present a solution based on RFID that sends rescue drawings and fire control data to a rescue team.

Table 7 presents a classification of the works mentioned in this section, taking into consideration their main objectives.

## 6. Other Applications in Interiors: Location and Navigation

As shown in previous sections, the use of RFID systems is really helpful during the erection of a building, furthermore the conception and installation of security devices in it. However, once the works are finished and the buildings inhabited, certain actions carried out in its interior can be controlled by means of RFID equipment.

### 6.1. Location and Mapping

One of the above-mentioned applications is the location of users and objects in inhabited interiors, as well as the generation of environment maps. In the last few years, many researchers have worked to solve indoor location sensing (ILS) problems with the help of radio-frequency identification technologies. Some of these techniques and their results have been evaluated and compared previously in [55].

Aiming to improve the accuracy of the existing systems and to avoid the addition of more tag readers, the LANDMARC location-sensing system [56] proposes the installation of some extra location reference active tags organized in a grid array to locate different objects inside a facility. Over the years, several works have improved this location system. Thus, the fusion of the LANDMARC and Bayesian-based algorithms [57] provides a new well-performed method. De Amorim Silva and Da S. Goncalves [58] improve the LANDMARC algorithm by means of an algorithm that provides a second estimation of the possible area in which an object could be located. Finally, the LANDMARC proposal is extended to evaluate the position, not only in the plane, but also in three dimensions by Khan and Antiwal [59].

These previous systems are centralized, implying that they require a power supply and a central server to calculate the position of the user. Aiming to decentralize the process and to avoid this dependence, a set of beacon nodes can be installed. These programmed wireless devices replicate a signature linked to the Received Signal Strength Indication (RSSI) and allow calculating the location of the mobile object [60].

Although the location of an object or a user in an environment by means of RFID technology is an application itself, this operation can be associated with other tasks. Thus, a user equipped with an RFID reader can reach a destination point in a tagged environment [61]. Furthermore, the evaluation of the user position inside a building can be useful to control the lighting of the rooms in an automatic manner [62] or even to interact with certain elements or services equipped with RFID systems.

On certain occasions, the element whose position is calculated is a mobile robot. Placing different tags in an inhabited environment and equipping the robot with two RFID antennas, the relative position of the mobile device can be calculated. If the robot is equipped with a laser scanner, a mapping process of the environment is carried out [63]. Once the map is generated, the robot can execute different tasks, like inspection and surveillance, acting as an autonomous security agent [64].

### 6.2. Navigation

Besides the location, RFID technology makes the navigation in inhabited interiors easier. The navigation of a user in an environment under special conditions (such as a fire or under construction) or even an unknown one should be emphasized.

In the same manner, blind or visually-impaired people may have important troubles moving in unknown or changing environments. An example of a changing environment can be demonstrated in a grocery store. An RFID system may indicate certain easy navigation rules or even help to find the path to a useful destination [65,66]. In [66], RFID is also combined with Quick Response (QR) codes to facilitate the search for products. In addition to these applications, this system can be used as a tourist guide in a museum or a navigation system to help rescue teams in hazardous environments [67].

## 7. New Lines: Integration of RFID Systems with Different Technologies in Construction

The idea of combining technologies in construction has been consolidated in applications with RFID throughout the last four or five years. The use of RFID in this area facilitates an enormous variety of works. However, a tool can be much more useful and complete if different technologies are combined.

During the building process of a facility, from its conception to its finalization, many tasks must be supervised. In order to make these works easier and to improve their efficiency, they can be automatized by means of certain systems, which involves the use of different technologies. Nowadays, there exists a variety of publications in which several systems are combined. In the next subsections, we discuss two of the most common kinds of technology combinations based on RFID technology: personal digital assistants (PDAs) and computer vision.

Table 8 summarizes different solutions in which several technologies are combined with RFID.

### 7.1. RFID and PDAs

In a multitude of tasks, several users are required to interact with the RFID system. Therefore, the users are equipped with some sort of device that allows them to communicate with RFID targets located at large distances. In most cases, wireless devices and PDAs are used. The combination of these two different technologies is used over the lifecycle of a building, from its conception to its inhabitance.

Before starting the construction process, particularly during the fabrication of concrete components, RFID and PDAs technologies are combined [73]. The workers control the inspection tasks at the test labs and generate a portable data collection. Once the elements are manufactured, their management can be controlled. Thus, the information related to the inventory or the transport process can be sent and shared with the manager's office or the work site [26].

Another aspect to take into account is that the construction of new buildings has been decreasing in the last years in favour of the rehabilitation of inhabited facilities [75]. Therefore, there exist buildings whose components can be reused or changeable. These buildings are called open buildings [76]. The management of the elements that are part of this kind of building has been carried out by means of an RFID and PDA system in [51] to help architects and engineers to reanalyse and redesign the building's components.

Once the building is inhabited, there exists a variety of components that need maintenance tasks. These elements can be part of the structure of the building or equipment. The installation of RFID tags in these components allows the management of information related to the elements that need to be repaired or verified in the building. Therefore, a scheduling process can be developed in order to organize the different tasks [52,74] (see Section 5).

As mentioned before, RFID technologies can be useful to control and prevent disasters in buildings. Sending information related to the scene where a fire breaks out, by means of a PDA, can save precious time in rescue operations [54].

### 7.2. RFID and Vision

Aiming to register the advance of a construction project, a scheduling and budget control model can be generated [45]. The implementation of barcodes or RFID systems allows controlling the labour force, material and tool location. A complete representation of the site under construction can be carried out by means of laser scanners and photogrammetry. In this manner, possible defects in constructive components can be detected [68]. Finally, the 3D models generated from the data acquired by means of the laser scanner update the information stored in the plans and compare the planned and built models [69].

As mentioned in the previous section, during the lifecycle of a facility, several recognition or maintenance operations are carried out in different scenarios. Vision systems can be really useful in these works. However, if the sensed objects are not completely visualized, the task of object recognition could become very complex. In these cases, a set of RFID tags can provide certain information about the elements making the recognition easier. The information acquired from the RFID tags allows knowing what objects, among those stored in a database, are present in the scenario under study [70].

Vision systems can be composed by cameras, which provide bidimensional images in which several object parameters are extracted [71] or laser scanners, which give 3D information of the scene [70,72].

#### 7.2.1. A Novel Strategy: The Combination of RFID and Laser Scanning to Create BIM Models

An example of a current development in which RFID is introduced in construction is shown in this subsection. The aim here is to present RFID as a valuable tool in the field of automatic BIM.

As stated in previous sections, works in which RFID is involved are mainly oriented toward the building process, aiming to control the position of different resources in the job site. However, the use of this technology for monitoring inhabited sites or sites under construction has increased in the last few years, especially combined with other technologies (see Tables 6 and 8).

The system referred to in this subsection is based on the combination of RFID technologies and laser scanning to generate a 3D model of an inhabited environment. To date, many authors have tackled the generation of synthetic models of different kinds of facilities by means of terrestrial laser scanners (TLS), the automatic creation of these 3D representations being a cutting edge field of research in construction. However, data acquired by TLS consist of millions of unstructured 3D points that need to be treated.

**Figure 2:**
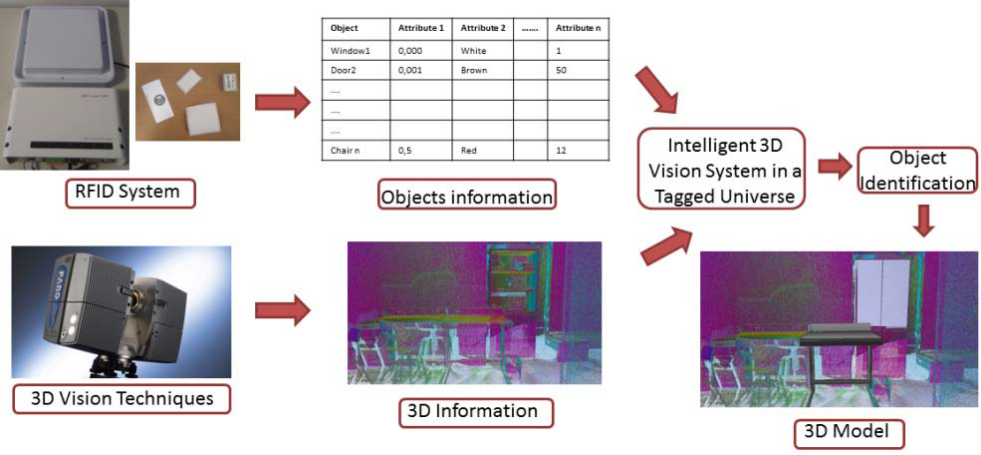
Overview of the system.

Aiming to alleviate the computing processes in this task, an RFID system is adapted to a laser scanner in [72]. As is demonstrated in this work, RFID provides essential information about the scene under study. This information, stored in RFID tags, is mainly related to the geometry of the basic pieces of furniture where tags are adhered. An overview of this system is shown in Figure 2.

**Figure 3:**
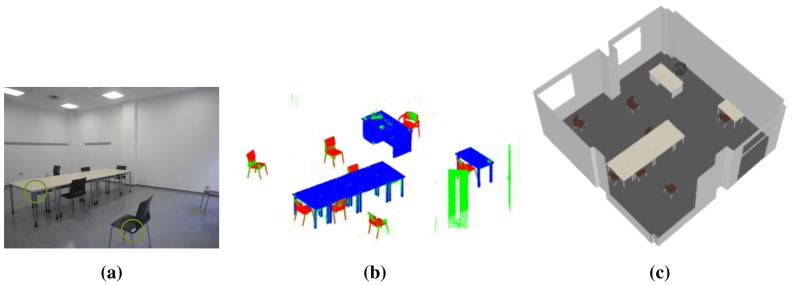
(**a**) Tags attached to pieces of furniture in an inhabited interior; (**b**) pieces of furniture identified and positioned in a point cloud; (**c**) 3D model of a classroom.

Once RFID and laser scanning operations are completed, the information acquired from the tags (see tags in Figure 3a) is merged with the 3D information to identify and position the pieces of furniture in the scene (see Figure 3b). Finally, a 3D model of the interior is generated (Figure 3c).

The system has been tested in different furnished scenarios and is able to identify and precisely pose in the scene common pieces of furniture, likes chairs, tables and wardrobes. While further details about the proposed system are not the object of this paper, the reader is referred to [72].

## 8. Discussion and Future Advances

From the conception of a building to its use, either as the only technology or combined with others, radio frequency identification technology has contributed to the automation of several works, improving their efficiency and reducing the associated costs. After reviewing the state-of-the-art in this framework, there exist several limitations and gaps that cause contractors to choose other available technologies.

Price: Although the price of UHF RFID systems has decreased, different solutions, such as near-field communication (NFC) or barcodes, are much less expensive [77].

Accuracy: Regarding the study of the location of different objects in the scene, the calculation of the position reached by RFID systems is not very accurate [78].

Interferences: Metals and concrete, very common in the field of construction, can cause some interferences during the information exchange process [17]. In fact, an additional surface must be placed between the tag and the object in many cases. Furthermore, reading problems can occur if tags are surrounded by metal.

Standardization: The different legislation existing worldwide about the use of RFID antennas can lead to some supposed difficulties in the trade of tagged components. Currently, the association EPCglobal is involved in the creation of standards for RFID, such as UHF Class 1 Generation 2 [79].

Concerning improvements and future research, different key aspects can be pointed out.

Building renovation: Looking ahead, the concept of open buildings [51] needed to be taken into account, considering the maintenance of facilities. This idea of renovating buildings instead of constructing new ones is being more and more considered nowadays [80].

Safety: One of the advantages of RFID is that the user does not need a direct view of a tag to identify it and get the stored information. Although there exist several works related to assistance in fires [67], this problem is not sufficiently tackled yet. One possible improvement of safety in buildings could be the control of constructive components, which are not accessible to users and need maintenance labour, like pipe spools or the electrical supply.

Building information management (BIM): This process, providing the physical and functional characteristics of facilities, is going to play a critical role in the transformation of the construction sector. As summarized in Subsection 7.2.1 and focused on this field of research, we have presented a system based on the combination of laser scanning and RFID, aiming to generate BIM models for inhabited interiors [72]. Furthermore, works based on RFID and related to the real-time location tracking of a user in a BIM model have been recently published [81,82].

Integration of RFID with other technologies: One of the stronger research lines in the future concerns the proposals related to the integration of RFID with personal digital assistants or other wireless devices, because of the rising market and the lowering of prices of this kind of gadget. As the reader can appreciate in this paper, most of the works developed in this field of research during the last five years have been focused on the combination of technologies, mainly in the monitoring of construction sites.

The communication between devices makes collaborative tasks and the sharing of information about works in progress or the maintenance of certain components easier. Therefore, new developments in the field of communication in construction integrating RFID based-technologies are expected in the next decade.

## Figures and Tables

**Table 1:** Operation bands for RFID technologies.

**Band**	**Frequency Range**	**Distance Range**	**Example Applications**
(125–150) kHz	Low frequency (LF)	<2 m	Animals ID
13.56 MHz	High frequency (HF)	<20 cm	Access and security
(433–928) MHz	Ultra-high frequency (UHF)	433–864 MHz <100 m	Logistics
		865–928 MHz <2 m	
(2.45–5.8) GHz	Microwave	<1 m	Mobile vehicle toll
(3–10.5) GHz	Ultra-wide band (UWB)	<10 m	(Early phases)

**Table 2:** Types of RFID transponders.

	**Active Tags**	**Passive Tags**	**Semi-Passive Tags**
**Distance range**	Up to 100 m	Up to 15 m	Up to 60–80 m
**Power**	Power supply (Battery)	Inducted from readers	Turned on by a signal
**Relative cost**	>30	1	>20
**Data storage**	Extendible and can vary	512 bytes to 4 KB	Extendible and can vary
**Data transfer rate**	Up to 128 KB/s	Up to 1 KB/s	Up to 16 KB/s
**Lifetime**	Up to 10 years	Unlimited	Over 6 years

**Table 3:** Works dealing with the initial stages of the construction process.

**Initial Stages**	**Material to Tag Interaction**	**RFID System Selection**	**Buried Systems**
[17]	✓	✓	
[18]	✓		
[19]	✓		
[20]	✓		
[21]		✓	
[22]		✓	✓

**Table 4:** Different solutions for manufacturing control with RFID.

**Manufacturing and Supply Chain**	**Concrete Operations**	**Precast Production Management**	**Pipe Spools**	**Transport and Delivery Control**	***CO*_2_ Emissions Control**
[24]	✓	Spools			
[25]	✓			✓	
[26]		✓		✓	
[28]				✓	
[29]				✓	✓
[27]			✓	✓	

**Table 5:** Use of RFID tags for locating resources.

**Tracking**	**Materials**	**Workers**	**Equipment**
[9]	✓		
[24]	✓	✓	✓
[32]	✓	✓	✓
[33]	✓		
[34]	✓		
[35]	✓		
[36]	✓		
[37]	✓		
[38]	✓		
[39]		✓	
[40]		✓	
[42]		✓	✓
[43]	✓	✓	✓

**Table 6:** Combination of technologies and RFID for construction site monitoring. BIM, building information management.

**Monitoring**	**Computer Vision**	**Barcodes**	**CAD/BIM**	**AR**	**GPS**	**GPRS**
[45]	✓	✓				
[46]			✓			
[47]			✓	✓		
[48]					✓	✓

**Table 7:** Works focused on the evaluation and maintenance of buildings.

**Evaluation and Maintenance**	**Drainage Systems**	**Open Buildings**	**Structural Components**	**Services and FM**	**Tasks Scheduling**	**Extinguishers/Fire Control**
[50]	✓					
[51]		✓	✓			
[52]				✓	✓	
[53,54]						✓

**Table 8:** Characteristics of different combinations of technologies.

**Reference**	**Accompanying Technology**	**Purpose**
[26]	PDA	Sending information to the manager's office or the site under construction
[45]	Laser scanner	Calculating quantities and site representation
	Photogrammetry	Calculating quantities and site representation
	Barcode	Collecting working hours
	CAD	Information/updating planned data
[68]	Laser scanner	Condition assessment
		Quality control defect detection
		History capture/Heritage
	Embedded sensors	Bridge footing inspection
		Building inspection
[51]	PDA	Maintenance management
[52]	Tablet PC	Data management, scheduling and data transfer between workers
[54]	PDA	Sending drawings and information to the rescue teamin fires
[69]	Camera	Image recognition
	Mobile computing	Communication support and information consistency checking
[70]	Laser scanner	3D information from the scene
[71]	Camera	Initial pose estimation of objects
[72]	Laser scanner	3D information from the scene
[73]	PDA	Monitoring and control of inspection progress
[74]	PDA	Maintenance management

## References

[1] Aarts E., Appelo L. (1999). Ambient intelligence: Thuisomgevingen van de toekomst. IT Monit..

[2] Aarts E. (2004). Ambient intelligence: A multimedia perspective. IEEE Multimed..

[3] Lindwer M., Marculescu D., Basten T., Zimmermann R., Marculescu R., Jung S., Cantatore E. Ambient Intelligence Visions and Achievements: Linking abstract ideas to real-world concepts.

[4] Calis G., Deora S., Li N., Becerik-Gerber B., Krishnamachari B. Assessment of WSN and RFID Technologies for Real-Time Occupancy Information.

[5] Yan Q. (2015). Research on Fresh Produce Food Cold Chain Logistics Tracking System Based on RFID. Adv. J. Food Sci. Technol..

[6] Mennecke B., Townsend A. (2005). Radio Frequency Identification Tagging as a Mechanism of Creating a Viable Producer's Brand in the Cattle Industry.

[7] Dalton J., Ippoilto C., Poncet I., Rossini S. (2005). Using RFID Technologies to Reduce Blood Transfusion Errors, White Paper.

[8] Amini M., Otondo R., Janz B., Pitts M. (2007). Simulation Modeling and Analysis: A Collateral Application and Exposition of RFID Technology. Product. Oper. Manag..

[9] Yagi J., Arai E., Arai T. (2005). Parts and packets unification radio frequency identification (RFID) application for construction. Autom. Constr..

[10] Thiesse F., Dierkes M., Fleisch E. (2006). LotTrack: RFID-Based Process Control in the Semiconductor Industry. IEEE Pervasive Comput..

[11] Lu W., Huang G.Q., Li H. (2011). Scenarios for applying RFID technology in construction project management. Autom. Constr..

[12] Ergen E., Akinci B. An Overview of Approaches for Utilizing RFID in Construction Industry.

[13] Erabuild (2006). Review of the Current State of Radio Frequency Identification (RFID) Technology, Its Use and Potential Future Use in Construction.

[14] Dobkin D.M. (2012). The RF in RFID: UHF RFID in Practice.

[15] Klair D.K., Chin K.W. (2010). A Survey and Tutorial of RFID Anti-Collision Protocols. IEEE Commun. Surv. Tutor..

[16] Li N., Becerik-Gerber B. (2011). Life-Cycle Approach for Implementing RFID Technology in Construction: Learning from Academic and Industry Use Cases. J. Construct. Eng. Manag..

[17] Jaselskis E.J., El-Misalami T. (2003). Implementing Radio Frequency Identification in the Construction Process. J. Constr. Eng. Manag..

[18] Tzeng C.T., Chiang Y.C., Chiang C.M., Lai C.M. (2008). Combination of radio frequency identification (RFID) and field verification tests of interior decorating materials. Autom. Constr..

[19] Mo L., Zhang H. RFID Antenna Near the Surface of Metal.

[20] Zeng L., Grau D., Xiao Y. (2015). Assessing the Feasibility of Passive and BAP RFID Communications on Construction Site Scenarios. IEEE Syst. J..

[21] Chen S.L., Lin K.H., Mittra R. A low profile RFID tag designed for metallic objects.

[22] Dziadak K., Kumar B., Sommerville J. (2009). Model for the 3D Location of Buried Assets Based on RFID Technology. J. Comput. Civil Eng..

[23] van Gassel F., Jansen G. A Simulation Tool for Radio Frequency Identification Construction Supply Chains.

[24] Jaselskis E.J., Anderson M.R., Jahren C.T., Rodriguez Y., Njos S. (1995). Radio-Frequency Identification Applications in Construction Industry. J. Constr. Eng. Manag..

[25] Moon S., Yang B. (2010). Effective Monitoring of the Concrete Pouring Operation in an RFID-Based Environment. J. Comput. Civil Eng..

[26] Yin S.Y., Tserng H.P., Wang J., Tsai S. (2009). Developing a precast production management system using RFID technology. Autom. Constr..

[27] Song J., Haas C.T., Caldas C., Ergen E., Akinci B. (2006). Automating the task of tracking the delivery and receipt of fabricated pipe spools in industrial projects. Autom. Constr..

[28] Grau D., Zeng L., Xiao Y. (2012). Automatically tracking engineered components through shipping and receiving processes with passive identification technologies. Autom. Constr..

[29] Kaneko T., Hamada K., Kondo T. Development of Construction Logistics System Using Radio Frequency Identification.

[30] Thomas H.R., Sanvido V.E., Sanders S.R. (1989). Impact of Material Management on Productivity—A Case Study. J. Constr. Eng. Manag..

[31] Bjork B.C. (1992). A unified approach for modelling construction information. Build. Environ..

[32] Su X., Li S., Yuan C., Cai H., Kamat V. (2014). Enhanced Boundary Condition—Based Approach for Construction Location Sensing Using RFID and RTK GPS. J. Constr. Eng. Manag..

[33] Cai H., Andoh A.R., Su X., Li S. (2014). A boundary condition based algorithm for locating construction site objects using {RFID} and {GPS}. Adv. Eng. Inform..

[34] Song J., Haas C.T., Caldas C.H. (2006). Tracking the Location of Materials on Construction Job Sites. J. Constr. Eng. Manag..

[35] Skibniewski M.J., Jang W.S. Localization Technique for Automated Tracking of Construction Materials Utilizing Combined RF and Ultrasound Sensor Interfaces.

[36] Kim C., Ju Y., Kim H., Kim J. Resource Management in Civil Construction Using RFID Technologies.

[37] Huang R.Y., Tsai T.Y. Development of an RFID System for Tracking Construction Residual Soil in Taiwan.

[38] Zhang L., Atkins A. (2015). A Decision Support Application in Tracking Construction Waste Using Rule-based Reasoning and RFID Technology. Int. J. Comput. Intell. Syst..

[39] Chae S., Kano N. A Location System with RFID Technology in Building Construction Site.

[40] Goodrum P.M., McLaren M.A., Durfee A. (2006). The application of active radio frequency identification technology for tool tracking on construction job sites. Autom. Constr..

[41] The Center for Construction Research and Training (2013). The Construction Chart Book.

[42] Chae S. Development of Warning System for Preventing Collision Accident on Construction Site.

[43] Wu W., Yang H., Li Q., Chew D. (2013). An integrated information management model for proactive prevention of struck-by-falling-object accidents on construction sites. Autom. Constr..

[44] Yoon S., Chin S., Kim Y., Kwon S. An Application Model of RFID Technology on Progress Measurement and Management of Construction Works.

[45] El-Omari S., Moselhi O. (2011). Integrating automated data acquisition technologies for progress reporting of construction projects. Autom. Constr..

[46] Montaser A., Moselhi O. (2013). RFID indoor location identification for construction projects. Autom. Constr..

[47] Wang X., Love P.E., Kim M.J., Park C.S., Sing C.P., Hou L. (2013). A conceptual framework for integrating building information modelling with augmented reality. Autom. Constr..

[48] Liu D., Cui B., Liu Y., Zhong D. (2013). Automatic control and real-time monitoring system for earthrock dam material truck watering. Autom. Constr..

[49] Josephson P.E., Hammarlund Y. (1999). The causes and costs of defects in construction A study of seven building projects. Autom. Constr..

[50] Kondo T., Uchida S., Kaneko T., Hamada K., Miyaura S., Okura M. Development of RFID-Based Flow Examination System.

[51] Cheng M., Lien L., Tsai M., Chen W. Open-Building Maintenance Management Using RFID Technology.

[52] Ko C.H. (2009). RFID-based building maintenance system. Autom. Constr..

[53] Motamedi A., Hammad A. RFID-Assisted Lifecycle Management of Building Components Using BIM Data.

[54] Shiau Y., Tsai J., Cheng S. Fire Control in Buildings and the Development of RFID Applications Systems.

[55] Li N., Becerik-Gerber B. (2011). Performance-based evaluation of RFID-based indoor location sensing solutions for the built environment. Adv. Eng. Inform..

[56] Ni L., Liu Y., Lau Y.C., Patil A. (2004). LANDMARC: Indoor location sensing using active RFID. Wirel. Netw..

[57] Huang Y., Liu Z., Ling G. An Improved Bayesian-Based RFID Indoor Location Algorithm.

[58] De AmorimSilva R., Da S., Goncalves P.A. Enhancing the Efficiency of Active RFID-Based Indoor Location Systems.

[59] Khan M., Antiwal V. Location Estimation Technique using Extended 3-D LANDMARC Algorithm for Passive RFID Tag.

[60] Lorincz K., Welsh M. (2007). MoteTrack: A robust, decentralized approach to RF-based location tracking. Pers. Ubiquitous Comput..

[61] Pradhan A., Ergen E., Akinci B. (2009). Technological Assessment of Radio Frequency Identification Technology for Indoor Localization. J. Comput. Civ. Eng..

[62] Zhen Z.N., Jia Q.S., Song C., Guan X. An Indoor Localization Algorithm for Lighting Control using RFID.

[63] Joho D., Plagemann C., Burgard W. Modeling RFID signal strength and tag detection for localization and mapping.

[64] Milella A., Cicirelli G., Distante A. (2008). RFID-assisted mobile robot system for mapping and surveillance of indoor environments. Ind. Robot Int. J..

[65] Kulyukin V., Gharpure C., Nicholson J. RoboCart: Toward robot-assisted navigation of grocery stores by the visually impaired.

[66] Lopez-de-Ipina D., Lorido T., Lopez U. (2011). Indoor navigation and product recognition for blind people assisted shopping. Ambient Assisted Living.

[67] Chumkamon S., Tuvaphanthaphiphat P., Keeratiwintakorn P. A blind navigation system using RFID for indoor environments.

[68] Kiziltas S., Akinci B., Ergen E., Tang P. (2008). Technological assessment and process implications of field data capture technologies for construction and facility/infrastructure management. ITcon.

[69] Rebolj D., Babič N.U., Magdič A., Podbreznik P., Pšunder M. (2008). Automated construction activity monitoring system. Adv. Eng. Inform..

[70] Cerrada C., Salamanca S., Adan A., Perez E., Cerrada J.A., Abad I. (2009). Improved Method for Object Recognition in Complex Scenes by Fusioning 3-D Information and RFID Technology. IEEE Trans. Instrum. Meas..

[71] Hontani H., Nakagawa M., Kugimiya T., Baba K., Sato M. A Visual Tracking System Using an RFID-tag.

[72] Valero E., Adan A., Cerrada C. (2012). Automatic Construction of 3D Basic-Semantic Models of Inhabited Interiors Using Laser Scanners and RFID Sensors. Sensors.

[73] Wang L.C. (2008). Enhancing construction quality inspection and management using RFID technology. Autom. Constr..

[74] Cong Z., Mo K., Menzel K. Development of a RFID-Based Building Maintenance System for facility management.

[75] Green B. (2012). Renovation and Green Agenda Support Weak Construction Activity across Europe. http://brickonomics.building.co.uk/2012/06/renovation-and-green-agenda-support-weak-construction-activity-across-europe/.

[76] Kendall S., Teicher J. (2000). Residential Open Building.

[77] Bravo J., Hervas R., Chavira G., Nava S., Villarreal V. From implicit to touching interaction: RFID and NFC approaches.

[78] Jang W.S., Skibniewski M.J. Wireless Network-Based Tracking and Monitoring on Project Sites of Construction Materials.

[79] (2013). EPC Radio-Frequency Identity Protocols Generation-2 UHF RFID.

[80] Sakamoto S., Kano N., Igarashi T., Tomita H. Laser Positioning System Using RFID-tags.

[81] Guo H., Liu W., Zhang W., Skitmor M. A BIM-RFID Unsafe On-Site Behavior Warning System.

[82] Costin A., Pradhananga N., Teizer J. Passive RFID and BIM for Real-time Visualization and Location Tracking.

